# Non-iridescent yet angle-dependent structural colors on titanium surfaces induced by laser oxidation

**DOI:** 10.1515/nanoph-2025-0149

**Published:** 2025-07-01

**Authors:** XiaoSong Yu, MingYang Wang, QiLin Jiang, ChenHui Lu, TianLi Feng, Jiao Geng, LiPing Shi

**Affiliations:** Hangzhou Institute of Technology, Xidian University, 311200, Hangzhou, China; School of Mechanical and Automotive Engineering, Shanghai University of Engineering Science, 201620, Shanghai, China; China Key Laboratory of Laser & Infrared System (Ministry of Education), Shandong Provincial Key Laboratory of Laser Technology and Application, School of Information Science and Engineering, Shandong University, 266237, Qingdao, China; School of Optoelectronic Engineering, Xidian University, 710071, Xi’an, China

**Keywords:** structural colors, laser nanofabrication, optically variable, titanium

## Abstract

Optically variable features are widely used in product design and anti-counterfeiting. However, current industrial methods rely heavily on chemical inks, which pose environmental concerns and suffer from poor wear and corrosion resistance. We experimentally demonstrate the generation of non-iridescent yet angle-dependent structural colors on titanium surfaces using a nanosecond laser-induced oxidation. Unlike conventional optical color-change methods that rely on multilayer interference, grating diffraction, or surface plasmons, this technique leverages a periodically arranged stepped structure to achieve abrupt color changes under small angle variations. The color shift originates from morphological differences among structures at different heights, which reflect light at distinct angles and produce varying colors through interference effects. The formation mechanism is elucidated through numerical simulations of the processing temperature, revealing that controlled laser ablation, oxidation, and thermal radiation on the sample surface create the unique structure. By tuning the point distance and dwell time, the affected area and intensity of these processes can be regulated. This advancement not only provides new ideas for anti-counterfeiting applications but also broadens the capabilities of laser coloring technology.

## 1 Introduction

As a unique decorative design, color-changing patterns are more attractive to consumers than single-color patterns. Common color change technologies include thermochromic [1], photochromic [2], optically variable color [3], etc. Among optically variable color, the color dependent on the viewing angle has become an important means of anti-counterfeiting [4] for banknotes, confidential documents, and products due to the color change that can be achieved by changing the viewing angle in natural light [5].

Surface color hue alterations in modern printing employ ink-based or ink-less methods. These changes manifest themselves as continuous transitions (rainbow hues) or abrupt mutations [6], [7], [8]. The core mechanisms involve multilayer thin film interference, grating diffraction, and localized magnetic surface plasmon polaritons (LMSPP) [9], [10], [11]. Ink-based systems achieve color shifts through interference effects by embedding particles in pigments or multi-coating [12], [13]. Three decades ago, Phillips et al. demonstrated a sharp color mutation at wide viewing angles using vacuum-rolled multilayer films. Subsequent work eliminated coatings by grinding the materials into pigments for color-shifting prints [14]. In recent years, photonic crystal based optically variable inks have garnered significant interest. The periodic arrangement of multilayer layers in photonic crystals results in intricate light interference that influences color reproduction [15]. Wang et al. have made research by mixing acrylic acid, acrylamide, and olefin monomers to make a rapidly curing photonic crystal condensation ink. The inked surface can exhibit color variation under a significant angle change of view [16].

In inkless printing, the majority of color-changing effects are achieved by LMSPP and grating diffraction [17], [18]. Si et al. fabricated a metasurface structure on a silicon substrate using the periodic arrangement of gold nano-rods. They utilized the LMSPP principle to develop a surface that exhibits four distinct colors: red, yellow, blue, and green, when observed from various angles [19]. In grating diffraction, Yang et al. used a femtosecond pulsed laser in a nitrogen environment to induce laser-induced periodic surface structures (LIPSS) on a silicon wafer. This method produced rainbow colors at various angles [20]. Geng et al. used femtosecond lasers to create helical LIPSS structures on amorphous silicon, resulting in a ring-shaped structure composed of radial color bands [21]. Building on this approach, the researchers added waveguide materials and observed a guided-mode resonance (GMR) phenomenon [22]. Whereby Koirala et al. constructed an ultrathin one-dimensional aluminum grating structure on silicon nitride, achieving a polarization-controlled color change effect [23].

Ink, as a mixture of various chemicals, inevitably presents several issues, including high environmental pollution [24], toxic gas emissions, and poor abrasion resistance [25]. In inkless printing, aiming for non-rainbow color effects, often relies on constructing metasurfaces and using GMR or LMSPP [26]. However, these methods incur high time and capital costs [27], [28]. Such expenses make them unsuitable for large-scale industrial production. Therefore, there is a practical need for a process that is environmentally friendly, simple, and fast, while still enabling angle-dependent color changes. Laser marking technology is rapidly replacing traditional marking methods in industrial printing [29], [30], [31], [32]. It is widely used for QR codes, product nameplates, corporate logos, due to its non-contact, eco-friendly [29], and fast processing speed. Titanium has attracted attention since its discovery [33]. It is known for its high hardness, excellent biocompatibility, and other superior properties. Titanium surface oxides have high hardness and corrosion resistance [34], as well as excellent adhesion, which has led to a wide range of applications for titanium oxides in bone implants [35], photocatalysts [36] and gas sensors [37]. In addition, titanium has a wide range of applications in the jewelry industry [38], [39]. By adjusting the thickness of the oxide layer on the titanium surface, different colors can be produced. These properties enable the formation of colorful patterns on titanium surfaces by controlling the oxidation area and the thickness of the oxide layer. The resulting patterns maintain vibrant colors while also exhibiting high hardness [40]. In recent years, researchers have realized laser color marking on titanium by adjusting the laser parameters. Wang et al. used a nanosecond laser to form oxide layers with various colors on titanium surfaces by controlling the laser parameters and using the dynamic serpentine scanning (DSS) method. Subsequently, through secondary processing, the samples, whose brightness would have been weakened by the angle, achieved the effect that the color brightness did not vary with the angle [41]. Tahseen et al. used a nanosecond laser to oxidize a single point, i.e. static serpentine scanning (SSS), on the titanium surface by controlling the laser to achieve the color marking of highly complex images such as oil paintings, and the saturation and value of the color of the samples processed in this way would change with the angle and would be distorted [42]. In general, when marking titanium with a nanosecond laser, the surface color hue of the samples processed in the existing study does not change with the angle, regardless of whether it is DSS or SSS.

In this paper, we used a SSS process and optimized the laser parameters to form an oxide layer that switches between two colors depending on the viewing angle. We analyzed the microstructure and elemental distribution using optical microscopy, scanning electron microscope (SEM), X-ray energy dispersive spectroscopy (EDS) and confocal laser scanning microscope (CLSM). The characterization results show that the structure is different from all previous principles of optically variable color exhibited by metasurfaces. The formation mechanism is further explained by numerical simulations of the processing temperature. Besides, the pattern experiment verifies the feasibility of the process in industrial production.

## 2 Experimental details

**Figure 1:**
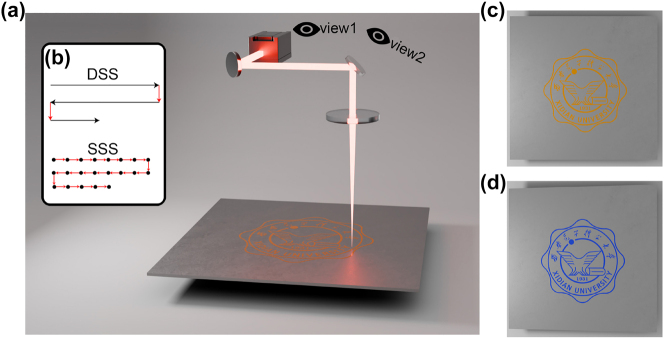
Schematic of (a) experimental setup and (b) processing methods; patterns under (c) view 1 and (d) view 2.

**In** our experiments, a 1 mm thick titanium plate with 99.9 % purity was cleaned with an ultrasonic cleaner and wiped with anhydrous ethanol prior to laser processing to ensure a clean surface. The experimental system is shown in Figure 1(a). The laser source used is a MOPA-configured fiber laser (JPT type YDFLP-E-20-M7-S-R), with a central wavelength of 1,064 nm, an output power of 20 W, a beam quality factor of *M* ^2^ = 1.4, a beam diameter of 7 mm, a pulse duration that can be adjusted within the range of 2–350 ns, and a frequency that can be adjusted from 1 to 4,000 kHz. The laser beam passes through a specular reflector and is focused on the titanium surface through an F-theta lens (SINO-GALVO type RC1001) with a focal length of 292 mm. The laser spot diameter is measured to be 40 μm at the focal point.

For laser coloring, the essence is that the laser acts as a generalized heat source and the processed material undergoes heat exchange, which produces a temperature field that causes physical and chemical changes in the center of the laser action and all around it, and roughly obeys a Gaussian distribution in the spatial distribution. The laser fluence can be expressed by the following equation: *F* = $\frac{4P}{f\pi D^{2}}$ [43], [44], where *P* denotes the average power, *f* the repetition rate and *D* the diameter of the focal spot.

Two commonly used laser color processing strategies are DSS and SSS methods [45], [46]. The trajectory of the laser in the two processing modes is shown in Figure 1(b). The DSS oxidation involves the laser continuously scanning across the sample surface. The SSS oxidation involves the laser to process discontinuous points intermittently. By controlling the laser dwell time, the total number of pulses on the sample is regulated. This method enables precise control of the degree of oxidation in the processed area. The formula for the total number of pulses during the dwell time in the SSS mode is *n* = *tf*, where *n* is the total number of pulses and *t* is the dwell time.

In addition, laser power, repetition rate, and pulse duration also affect the processing effect. In this paper, the SSS processing strategy is used to achieve the formation of an oxide layer with a color mutation effect on the titanium surface by controlling the processing parameters. Figure 1(c) presents the ideal color rendering effect of the sample observed from view 1 in Figure 1(a), corresponding to the front view. Figure 1(d) shows the ideal color rendering effect when the observation angle is shifted 14° to the right, corresponding to view 2 in Figure 1(a).

## 3 Results and discussion

**Figure 2:**
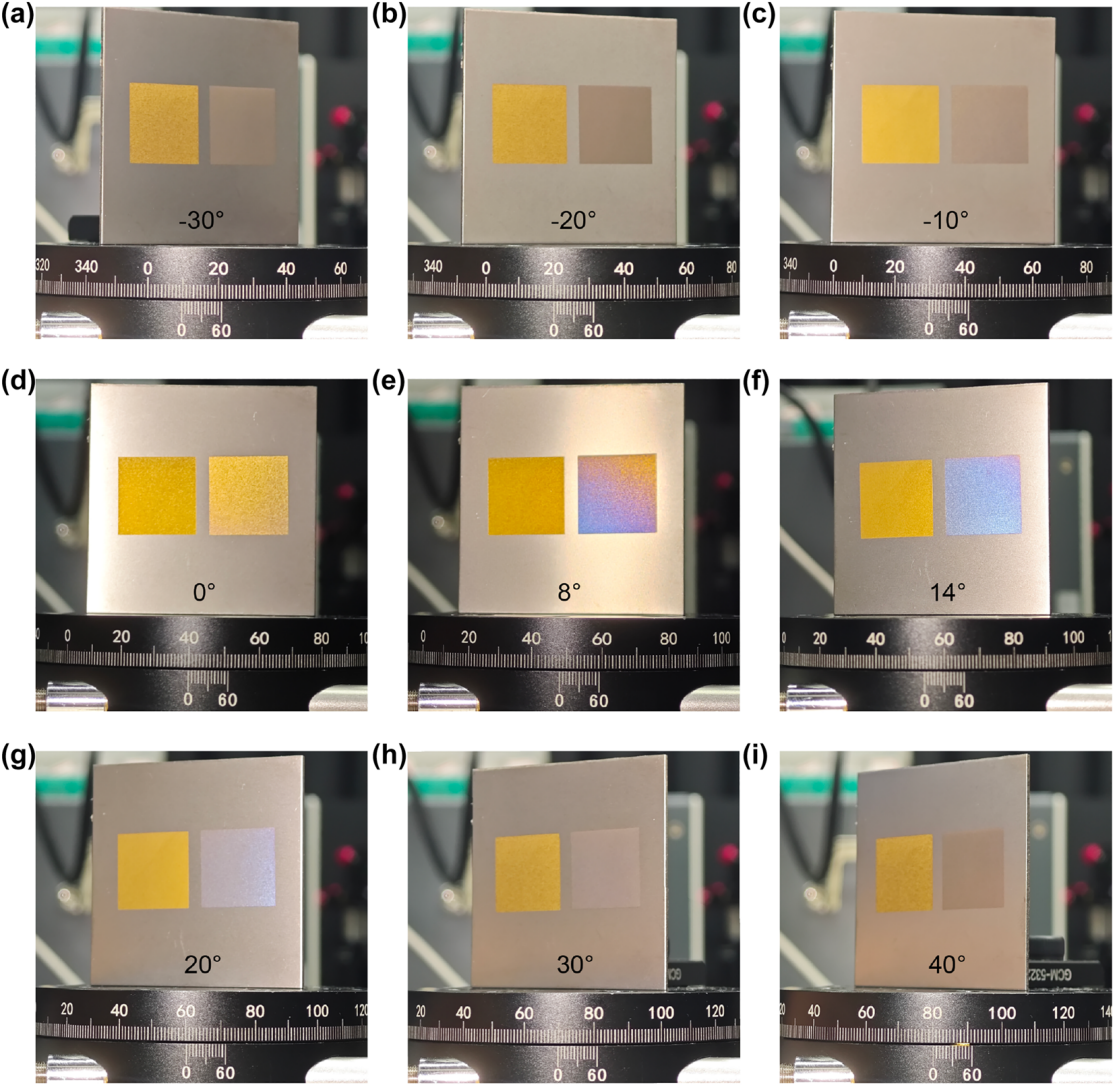
Comparison of DSS (left) processed and SSS (right) processed samples at (a) -30°, (b) -20°, (c) -10°, (d) 0°, (e) 8°, (f) 14°, (g) 20°, (h) 30°, (g) 40°.

Figure 2(a)–(h) show the color rendering effects of two samples processed according to different processing strategies at different illustrated rotation angles with the light source fixed and the same viewing position. For both samples, the laser settings remain identical. The laser settings include 50 % power, a 750 kHz repetition rate, and a pulse duration of 20 ns. The left sample was processed on a DSS with 50 μm line spacing and 300 mm/s sweep speed. The right sample was processed by SSS with 50 μm point distance and 0.9 ms standing time. Sample 1 shows a uniform yellow color at different angles, while Sample 2 is different from the common laser coloring samples, except for the yellow color under the front view, when the deflection angle of the sample continues to increase, the color of the sample will be gradually changed from yellow to blue, while the common coloring samples will only be limited to a single hue, and will not change hue with the change of angle (Color gamut of both at different angles in Figure S1 of the Supplementary Materials).

**Figure 3:**
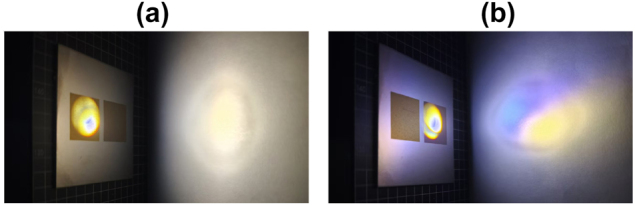
Color reflected from (a) DSS and (b) SSS processed sample under parallel light irradiation.

In order to illustrate this phenomenon more concretely, as shown in Figure 3, two samples appeared to have different reflection phenomena under illumination of parallel light in a dark environment. Figure 3(a) shows Sample 1, under parallel light irradiation, the reflected spot on the white board only shows yellow, while Figure 3(b) shows Sample 2, whose reflected spot consists of yellow and blue, and the separation of the two colors is obvious, which well illustrates that there are two different thicknesses of the oxide layer in Sample 2, and there is a difference in the refractive index of the two oxide layers to light.

**Figure 4:**
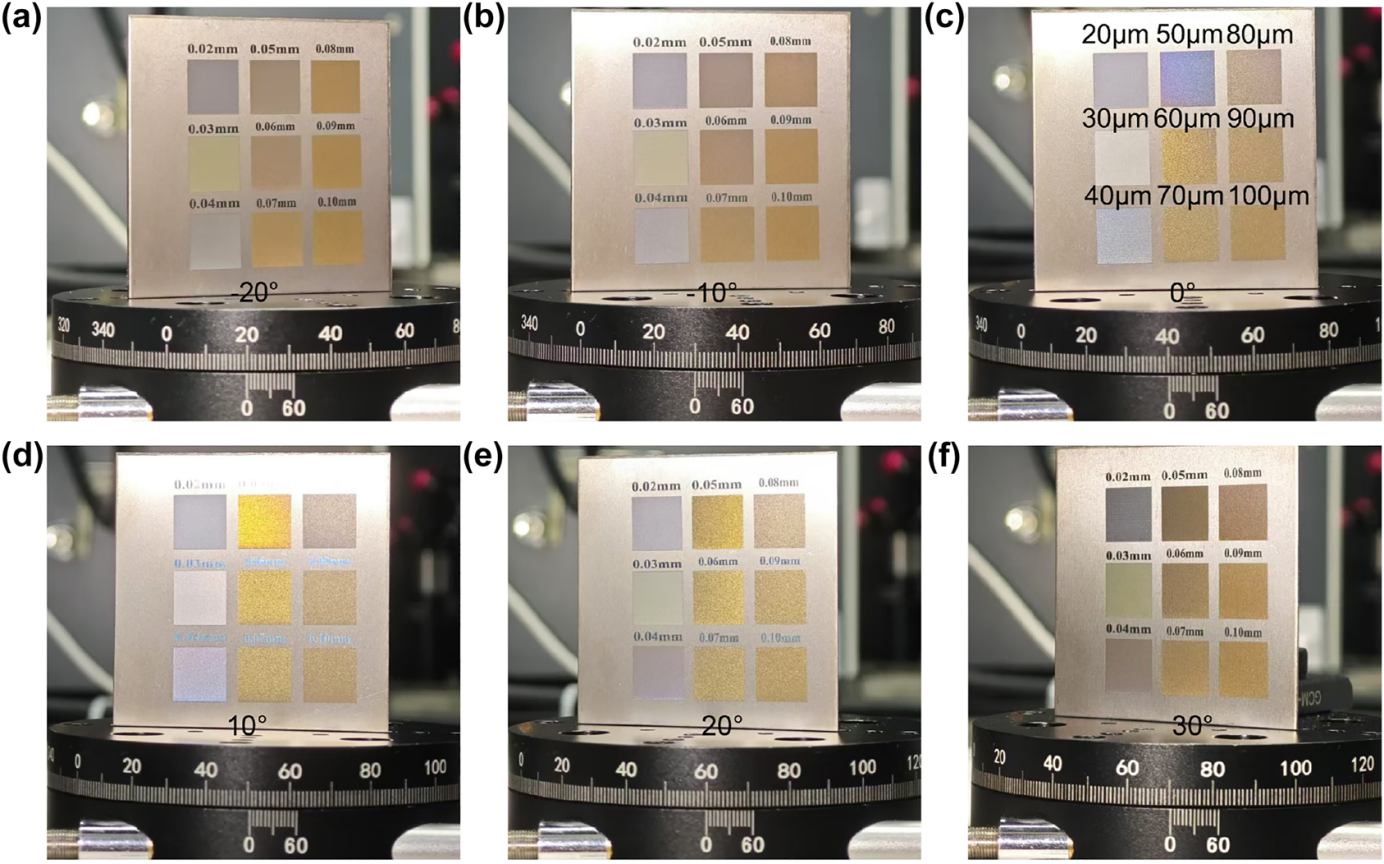
Photos of samples with different point distances at (a) -20°, (b) -10°, (c) 0°, (d) 10°, (e) 20°, (f) 30°.

As shown in Figure 4(a)–(f), we controlled the spacing of laser action points on the processed samples while keeping the laser parameters and dwell time constant. The point distance for each color block is indicated in Figure 4(c). Due to the different positions of the color blocks, the angle at which the color-shifting effect appears in Figure 4 differs from that in Figure 2. It is evident that all samples exhibited varying degrees of color change at different viewing angles. The most pronounced color shift occurred at a point distance of 50 μm. A slight change was observed at a spacing of 40 μm. Other color blocks showed no significant color variation. (Color gamuts of them at different angles in Figure S2(a)–(c) of the Supplementary Materials).

**Figure 5:**
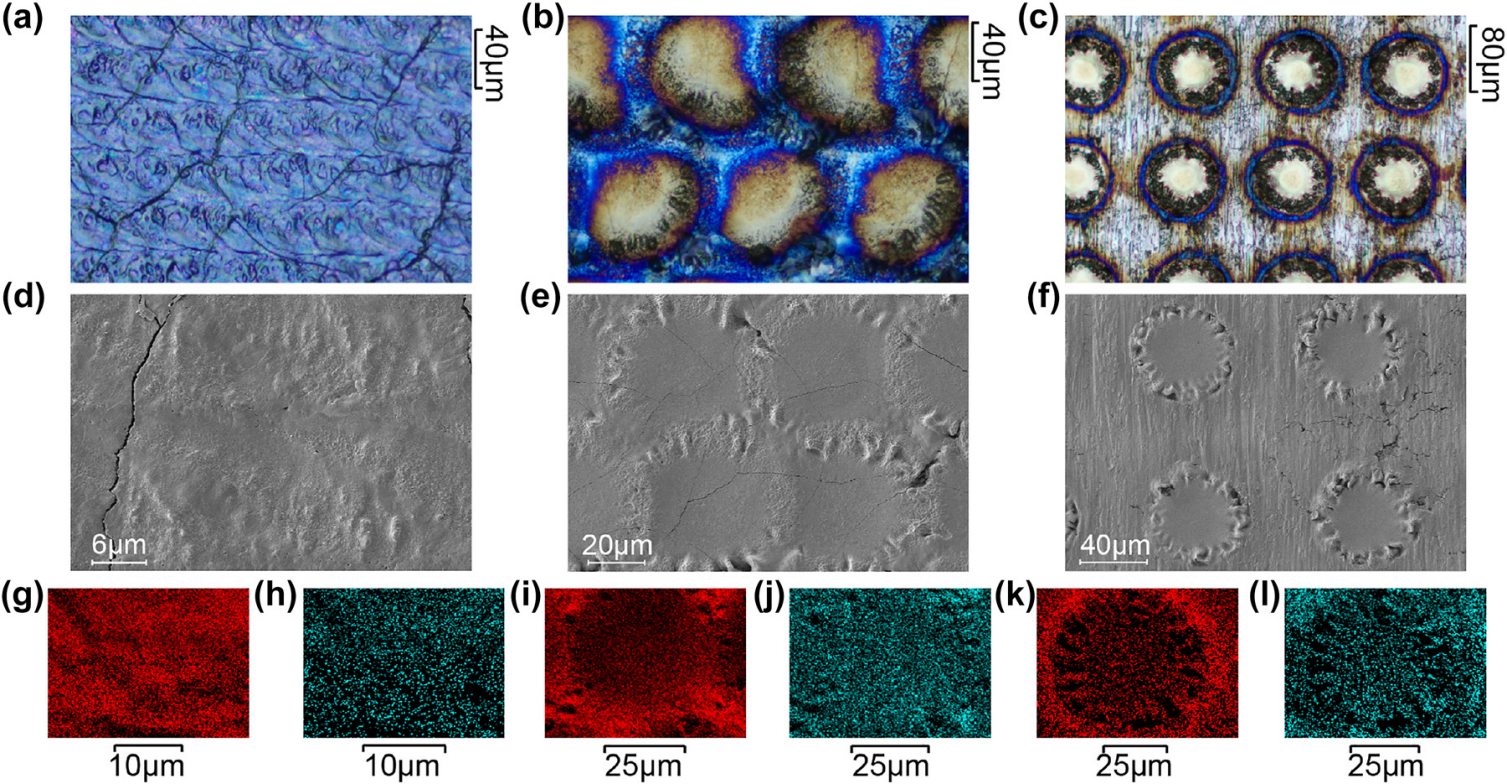
Optical microscope of samples with point distance of (a) 20 μm, (b) 50 μm and (c) 100 μm; SEM morphology of samples with point distances of (d) 20 μm, (e) 50 μm, and (f) 100 μm; Elemental oxygen distribution in samples with point distances of (g) 20 μm, (i) 50 μm and (k) 100 μm; Element distribution of oxygen in samples with point distance of (h) 20 μm, (j) 50 μm and (l)100 μm.

Here, samples with spot spacing of 20 μm, 50 μm and 100 μm were selected (the set spacing of 0.02 mm for Sample 3, 50 μm for Sample 4, and 100 μm for Sample 5). These three samples were further investigated microscopically, and the images of samples 3 and 4 under optical microscope are shown in Figure 5(a) and (b). In Sample 3 point distance equals spot radius. Thermal diffusion makes the processing similar to the sweeping-line method. The colors appear as a mixed light blue and pink-purple color. In the Sample 4 yellow blocks are arrange in order. The blue oxidized layers fill the gaps between the blocks. The spacing of the dots almost equals the diameter of the spot. Sequential processing causes later areas to affect earlier ones. Thus, yellow blocks are not perfectly circular. Because the distance between the dots is close to the diameter of the spot, and there is a sequential relationship in the processing order, the region processed after will have an impact on the previous processed region, so the yellow color block in the figure is not a standard circle. Figure 5(c) displays image of Sample 5 under optical microscope. The distance between the processing areas far exceeds the diameter of the point. Therefore, the effect of thermal diffusion between spots is small. Under this spacing, the processing area shows a standard round shape. The main body exhibits a yellowish hue. A tiny blue ring outlines the periphery, with a yellow diffusion band beyond (Optical microscope image of DSS processed sample in Figure S3 (a) of the Supplementary Materials).

In order to explore the morphology of the structures as well as the elemental distribution in more depth, three samples were photographed by SEM, analyzed by EDS and CLSM in this study (SEM image of DSS processed sample in Figure S3(b) of the Supplementary Materials). The cracks in the SEM images are fine traces caused by the internal stress release after the die-casting molding of the sample plates, which has almost no effect on the macroscopic color development of the samples [47]. Figure 5(d) shows the electron microscope image of the sample with a point distance of 3. At this point distance, there is no obvious geometrical partition in the micro-morphology of the sample, and the morphology of each place is generally consistent with the appearance of a disorderly distribution of small bumps, so that Sample 3 does not have a sudden change in color at different angles. Figure 5(e) displays Sample 4 under electron microscopy using a point distance. Different zones appear in Sample 4. The main effective region of the laser spot, shown in yellow in Figure 5(b), exhibits a smooth morphology. The outer ring of the circular pattern, shown as blue in Figure 5(b), is rough with obvious folds. These morphological differences result in varied light reflection angles between regions. Consequently, Sample 4 displays both yellow and blue colors when observed from different angles. Figure 5(f) shows Sample 5 under electron microscopy. Similarly to Sample 4, the laser-affected area is relatively smooth with an outer ring that has folds. However, the outer ring appears slightly bulged. The entire spot resembles a volcano crater. In the non-processed region, the original titanium morphology is retained. With this spacing, the thermal influence between processed areas is minimal. On a macroscopic scale, only the center of the spot appears yellow (more details of SEM images in Figure S4 of the Supplementary Information).

EDS, as one of the common elemental methods, can quickly reflect the elemental composition of the sample surface, so it is commonly used to analyze the elemental species and content of the micro-region composition of materials. As shown in Table 1, the structure of the test shows that after processing, the elements on the surface of the samples are mainly composed of *O*, *Ti* and *C*. presents the elemental percentages in three samples. As spot spacing decreases, the carbon content drops while the oxygen content increases significantly. This indicates that a smaller spot spacing leads to higher degree of oxidation.

**Table 1:** The standard enthalpy of generation (Δ*H* ^0^) as well as the standard entropy (*S* ^0^) of the components.

	*Ti* (%)	*O* (%)	*C* (%)
Sample 3	34.32	55.07	58.89
Sample 4	63.42	42.1	31.89
Sample 5	2.26	2.83	9.22

By selecting an area of the sample, the electron beam is moved back and forth across the sample surface. Each detected characteristic X-ray photon creates a bright spot on the display at the corresponding position. These spots represent the distribution of the element. By integrating them, an EDS mapping of the sample surface is generated [48]. Higher brightness indicates a higher element concentration. Figure 5(g), (i) and (k) show the distribution of oxygen elements in the three samples, it is easy to see that the distribution of oxygen elements in Sample 3 is more uniform, while the oxygen elements in Samples 4 and 5 are mainly distributed in the outer ring area of the spot, which is consistent with the optical microscope image of the structure above, because the blue oxide layer is thicker than the yellow oxide layer in the DSS laser processing, but it is confusing to find out the distribution of oxygen elements in Sample 4 and 5 intuitively, because the laser temperature distribution of oxygen elements is higher than the yellow oxide layer in Sample 3. Intuitively, the laser temperature distribution follows a Gaussian distribution, where the center temperature will be higher than the surrounding temperature, and higher temperatures can increase the intensity of oxidation [49]. Figure 5(h), (j) and (l) shows the distribution of titanium in three samples respectively, the distribution of titanium is relatively uniform in both samples 3 and 4, and there are a few black voids that are not very obvious in Sample 4, while obvious black voids appear in Sample 5. These voids form a circle in the spatial distribution, and the distribution of oxygen element in Sample 5, also appears the same black voids circle. These voids are generally caused by the presence of a large height drop in the region, which suggests that a phase transition may have occurred on the sample surface during the laser processing.

**Figure 6:**
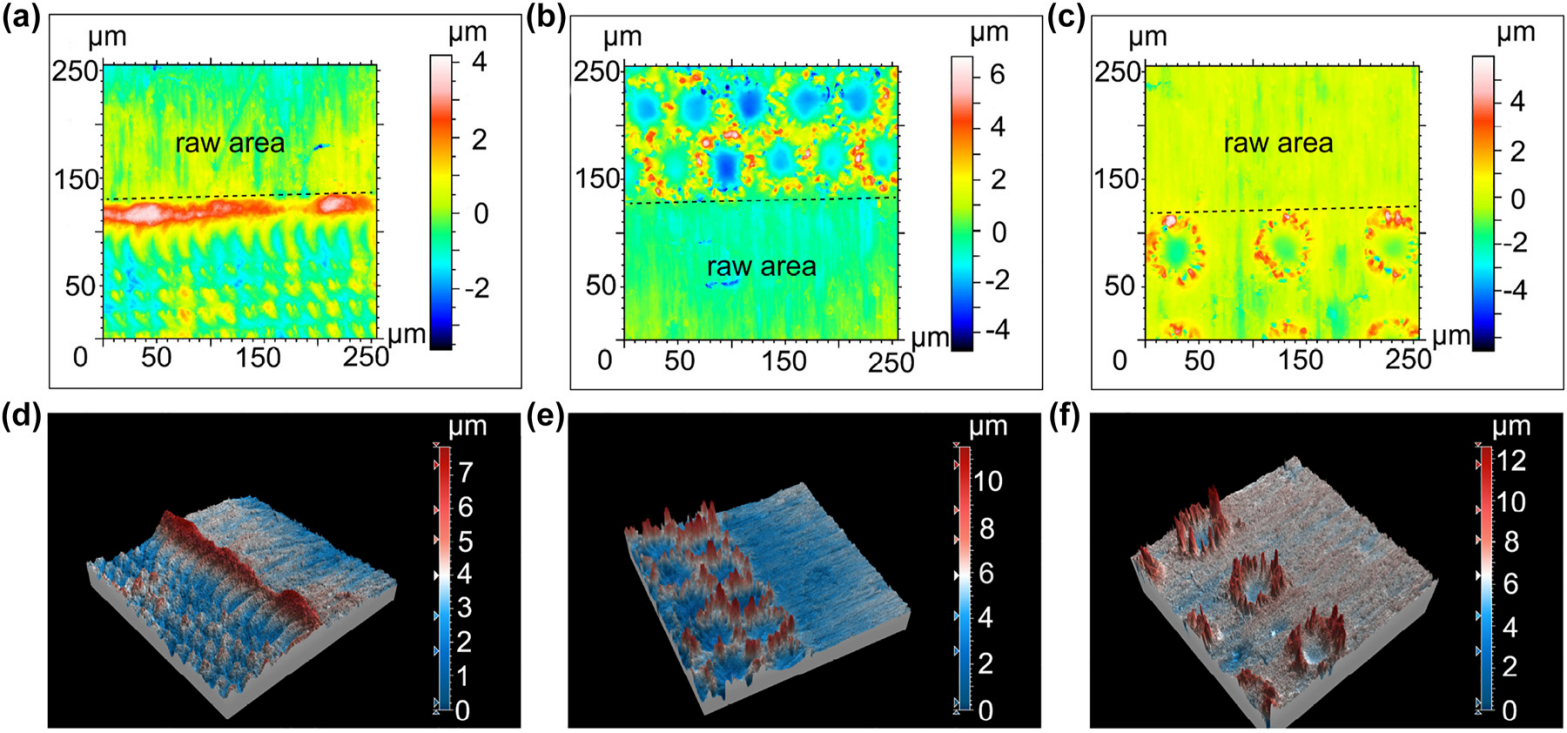
Two-dimensional height distribution on the sample surface at a point distance of (a) 20 μm, (b) 50 μm and (c) 100 μm; three-dimensional distribution on the sample surface at a point distance of (d) 20 μm, (e) 50 μm and (f) 100 μm.

To verify the above hypothesis, we used CLSM to characterize the surface height distribution of samples with point distances of 20 μm, 50 μm, and 100 μm. As shown in Figure 6(a) and (d), the processed region of Sample 3 shows a clear dot structure. A prominent bulge appears at the boundary between the processed and unprocessed areas, caused by melt extrusion during processing. From Figure 6(b) and (c), it is evident that Samples 4 and 5 both exhibit ring-like bulges in the processed region. However, only Sample 3 shows a clear negative height – i.e., a pit – in the center of the spot. This indicates severe ablation in Sample 4 at this pitch, while Sample 5 experiences milder ablation. Regarding the black voids mentioned earlier, Figure 6(e) and (f) reveal that the ring-shaped elevation around the spot periphery is not a smooth, regular circle. Instead, it resembles a mountain range, with alternating peaks and valleys. This observation supports the initial hypothesis.

**Figure 7:**
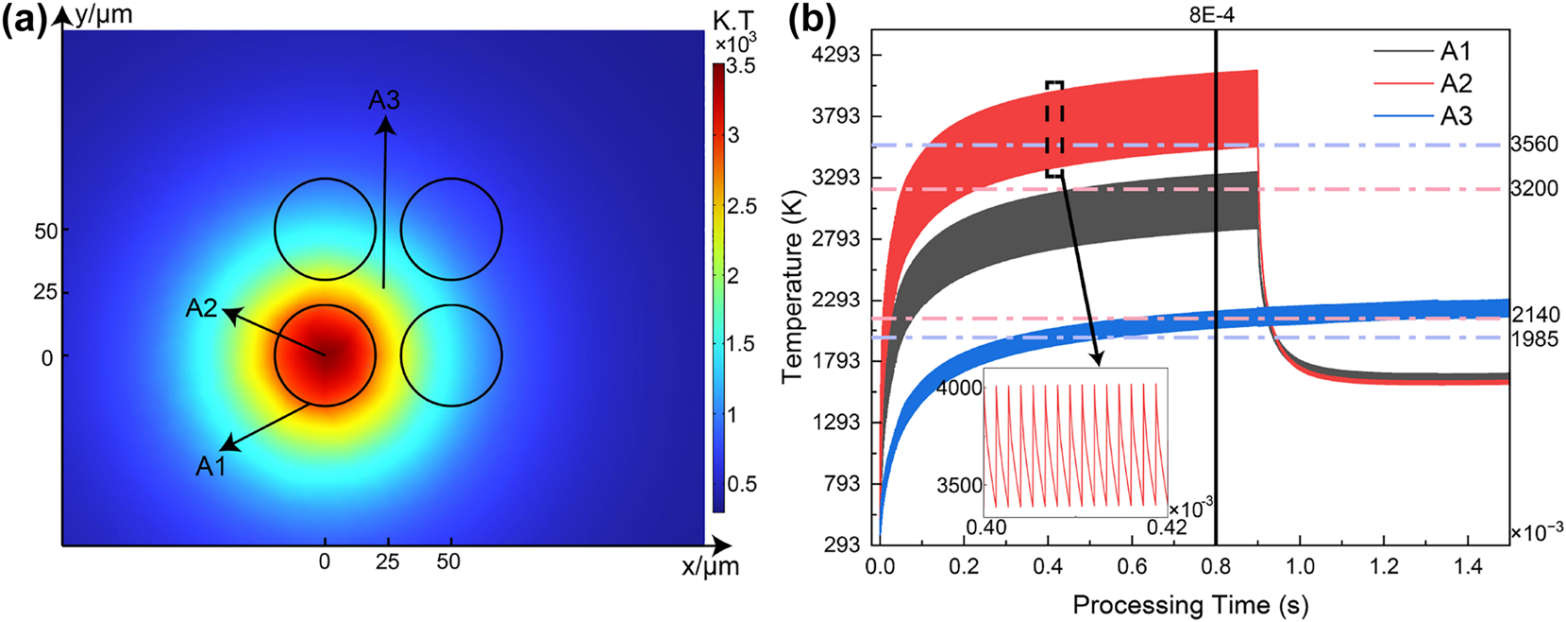
Numerical simulation of temperature during the oxidation process. (a) Spatial distribution of temperature at the end of oxidation at a single point; (b) temperature variation curves at different positions of the sample during processing.

In order to probe more deeply into the specific process of oxidation, COMSOL software was used to simulate the temperature distribution during laser processing at a 50 μm spacing. Figure 7(a) shows the temperature distribution at 0.9 ms, the total processing time for a single point. The temperature follows a Gaussian spatial distribution. Temperature probes were placed at different processing areas. A1 is the edge of the spot (defined as the edge of the focused laser spot, approximately 40 μm in diameter, rather than the processed region after laser exposure), A2 is the spot center, and A3 is the geometric center of four laser spots. Temperature variations over time are shown in Figure 7(b). Since pulsed laser processing was used, the laser power fluctuated over time, causing corresponding temperature fluctuations in the sample. According to reference data, the melting point of titanium is approximately 1,985 K and the boiling point is 3,560 K. The melting point of titanium dioxide is about 2,140 K, and the boiling point is 3,200 K [50]. Figure 7(b) shows that for most of the processing time, the center temperature of the spot exceeds the boiling point of titanium. At 0.8 ms dwell time, the center temperature completely exceeds this threshold. In contrast, the outer ring temperature does not exceed the boiling point of titanium. It only surpasses the boiling point of titanium dioxide for short periods. In the center of four points, the temperature exceeds the melting point of titanium dioxide from 0.8 ms onward. Once the dwell time is exceeded and the laser moves to the next point, the center and edge temperatures of the previous spot rapidly drop to around 1,650 K. However, the four-point center temperature remains stable.

**Figure 8:**
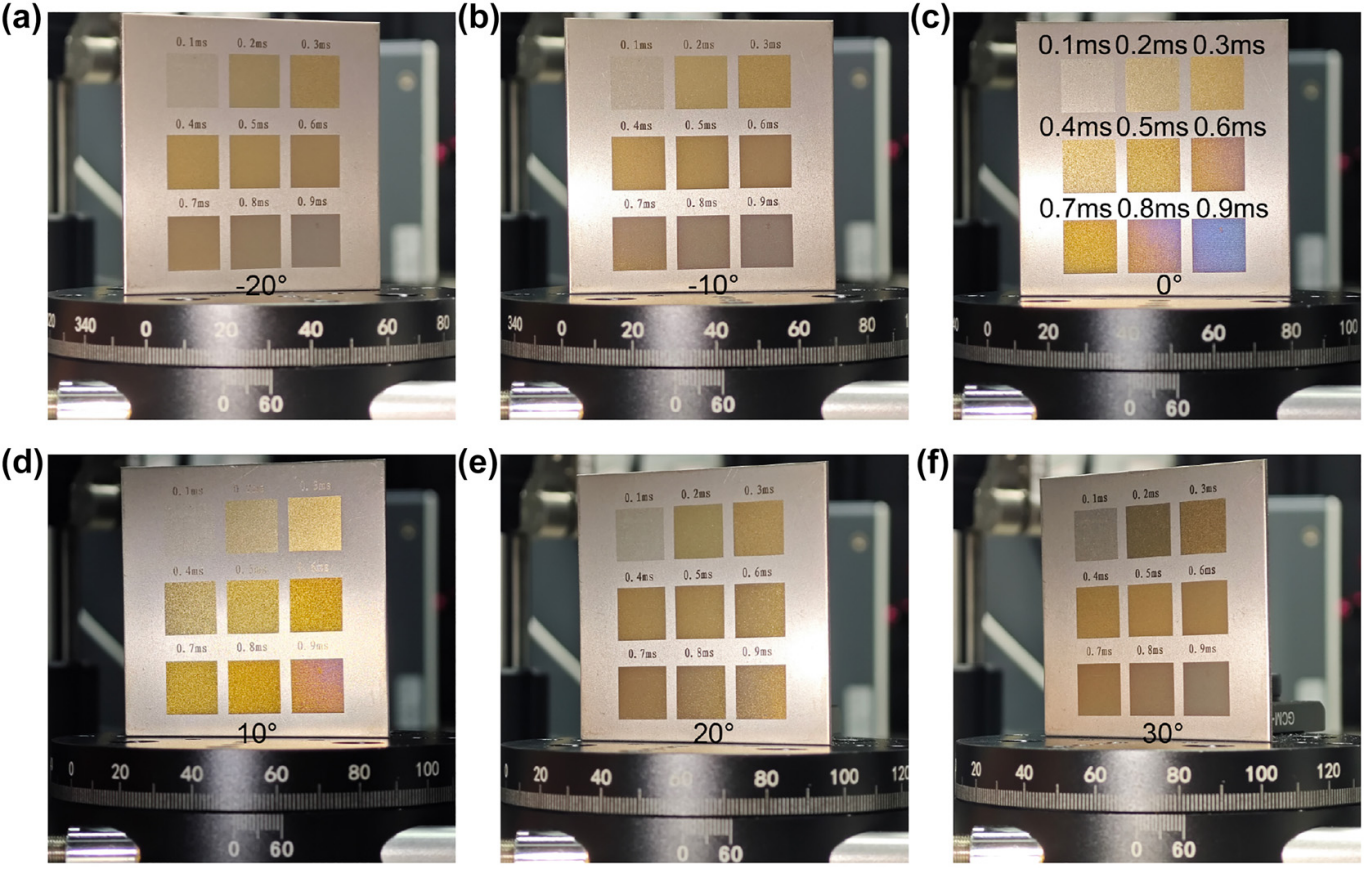
Color performance of samples at (a) -20°, (b) -10°, (c) 0°, (d) 10°, (e) 20°, (f) 30° for different dwell times.

Figure 8(a)–(f) shows the color variations of nine blocks at different viewing angles at various dwell times of lasers (Color gamuts of them at different angles in Figure S2(d)–(f) of the Supplementary Materials). The corresponding dwell times for each block are indicated Figure 8(c). The images reveal that only blocks with dwell times of 0.8 ms and 0.9 ms exhibit color shifts at different angles. All other blocks maintain consistent hues. The simulation results support this finding. Phase transitions of titanium and titanium dioxide are involved. Oxidation after laser exposure also plays a key role. These processes together lead to the observed color change.

Based on the EDS mapping of the three samples in Figure 5, molten titanium and titanium oxide flow outward under laser influence after reaching their melting points. As a result, the oxygen content at the center of the point in samples 4 and 5 is much lower than at the edges. Since Sample 4 has a smaller spot spacing than Sample 5, heat diffusion from the next laser pulse further affects the oxidation process of the previous spot. It is well known that when the temperature is higher than half of the melting point, intense oxidation of the metal begins [51]. Based on the simulation results in Figure 7(b), both the center of the spot and the outer ring continue to oxidize after processing. However, the ablation of titanium dioxide at the center is much greater than at the edges. As a result, the oxidation layers in the two regions differ significantly in thickness, leading to color variations. After laser exposure ends, the temperature at the spot center and edge rapidly drops below the melting points of titanium dioxide and titanium. The molten material from the center is pushed to the edges by the laser impact. Once the laser stops, both regions cool rapidly below their melting points. As a result, the spot interior remains smooth, whereas the edges develop a chaotic structure because of the irregular accumulation of molten material and rapid cooling. This difference in morphology creates a distinct contrast in the reflectivity between the center and the edge. In contrast, the temperature at the center of the four points remains above the melting points of titanium dioxide and titanium. The material remains in a liquid state for a longer period, allowing enough time for even diffusion. As a result, the morphology in the four-point center is much smoother than at the edges.

**Figure 9:**
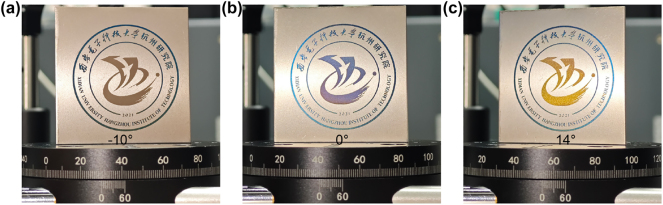
Color variations of the processed emblem at (a) -10°, (b) 0°, (C) 14°.

Figure 9(a)–(c) show the color variations of the processed emblem at different angles. By modifying the scanning strategy and replacing line filling with dot filling in specific areas, localized color changes can be achieved. Producing a 4 cm × 4 cm pattern like Figure 9 takes only 4 min, which makes the method suitable for large-scale industrial production.

## 4 Conclusions

In conclusion, we have created a periodic structure on the sample surface by controlling the laser for point-by-point processing. This structure consists of two distinct patterns with different thicknesses and shapes. Its unique design enables noticeable color changes at different angles, setting it apart from existing optical color change methods. Unlike existing optical color-change methods, this structure does not rely on multilayer interference, grating diffraction, or surface plasmons. Instead, a color change occurs because of variations in surface morphology. Micro-structured regions reflect light at different angles, and areas with different thicknesses produce distinct colors through interference effects.

Using optical microscopy, SEM, EDS, and CLSM, we reveal the specific effects of laser irradiation on the sample surface under the SSS method. By combining these results with numerical simulations of surface temperature, we gain a deeper understanding of the laser-induced oxidation process. The feasibility of the present processing in industrial production is further verified through the processing of complex patterns, which provides a new processing idea for the anti-counterfeiting process of optically variable. Future research can improve the process based on these findings, explore more color palettes, and expand the range of applicable materials.

## Supplementary Material

Supplementary Material Details
