# Supplementary material for: Non-iridescent yet angle-dependent structural colors on titanium surfaces induced by laser oxidation
Source: Nanophotonics. 2025 Jul 1;14(17):2869–79. doi: 10.1515/nanoph-2025-0149 (PMC12397728; doi:10.1515/nanoph-2025-0149)
Supplement: Supplementary file 1 — Supplementary Material Details [file j_nanoph-2025-0149_suppl_001.docx]

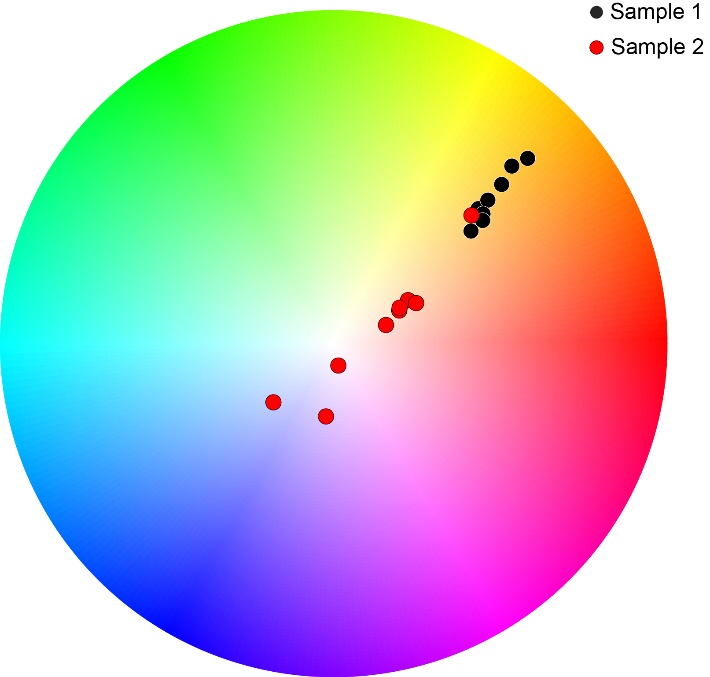


Fig. S1: Coordinates in HSV color space for DSS (Sample 1) and SSS (Sample 2) processed samples at different angles.

Figure S1 shows the HSV color space distribution at different viewing angles for samples processed by DSS and SSS methods. The hue and saturation of the DSS samples remain nearly constant across angles. In contrast, the SSS samples show significant variations. The hue shifts by more than 180° at certain angles, indicating a strong angle-dependent color change.


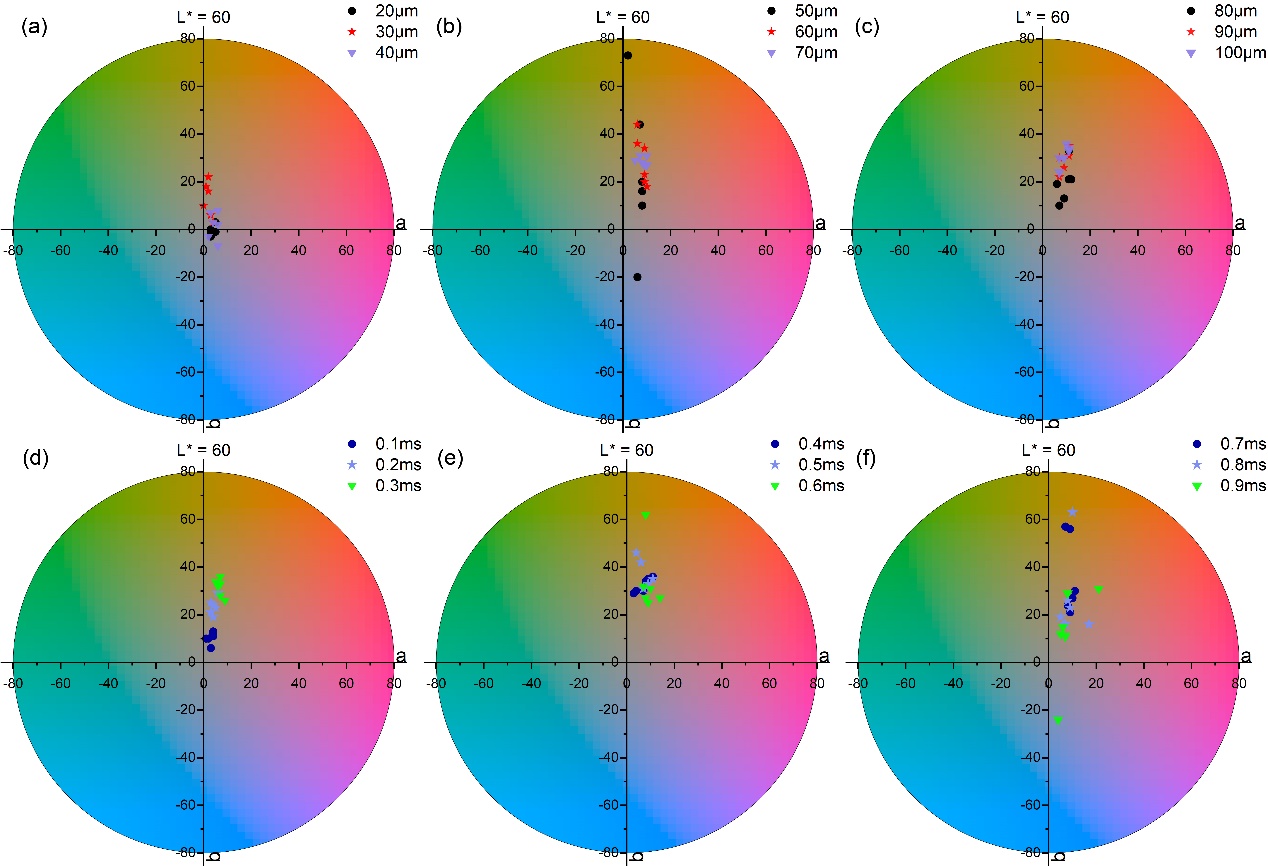


Fig.S2: Coordinates of colors in CIELAB at different angles for samples with different (a)-(c) point spacings and (d)-(f) stationing times

As shown in Fig. S2(a)–(c), regardless of the dot spacing, the *b* value of the sample color shows no significant change with viewing angle. However, when the dot spacing is 50 μm, the color exhibits a notable shift compared to other spacings, with the *b* value changing by up to 90 units. In Fig. S2(d)–(f), when the dot spacing is fixed and the dwell time is varied, an interesting trend appears. The maximum differences in both *a* and *b* values across viewing angles increase with dwell time. However, the change in *a* is much smaller than that in *b*.


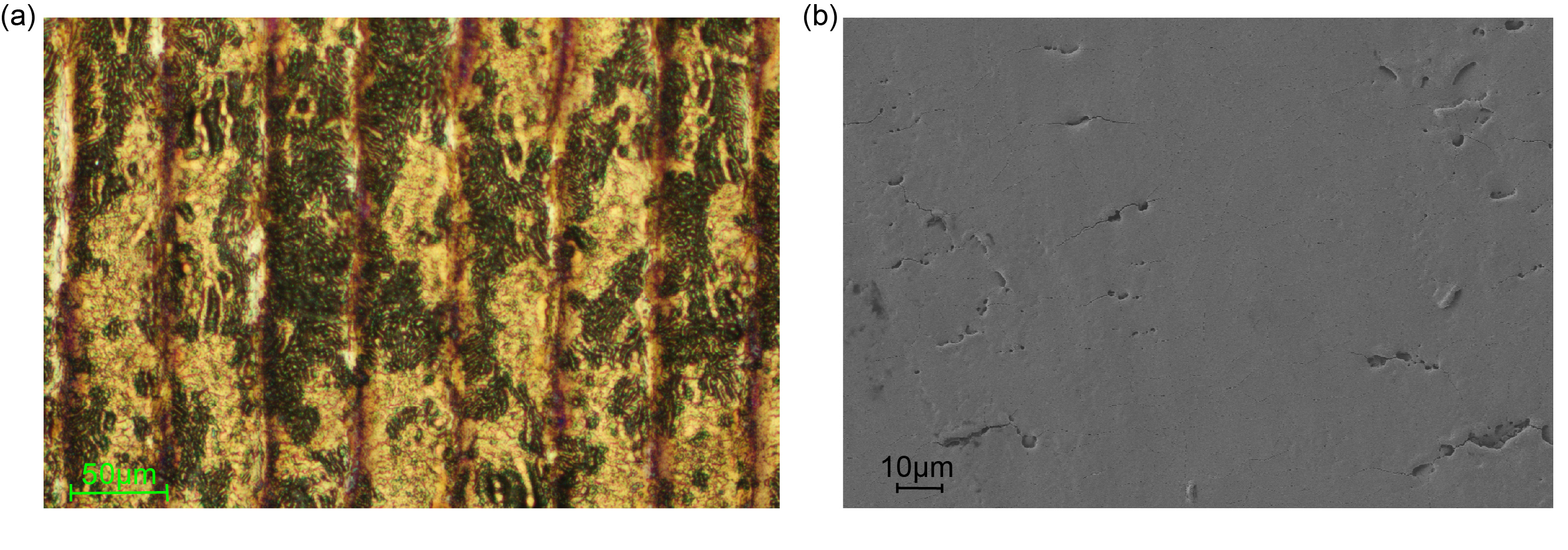


Fig.S3: (a) Image of Sample 1 under optical microscope; (b) SEM image of Sample 1.

As shown in Fig. S3(a), the samples processed by the DSS method display regular, stripe-like patterns under the light microscope and appear uniformly yellow. In Fig. S3(b), the microstructure of the DSS samples appears relatively flat. Upon closer inspection, faint striped features can be observed, which correspond to the brown lines seen in the optical image.


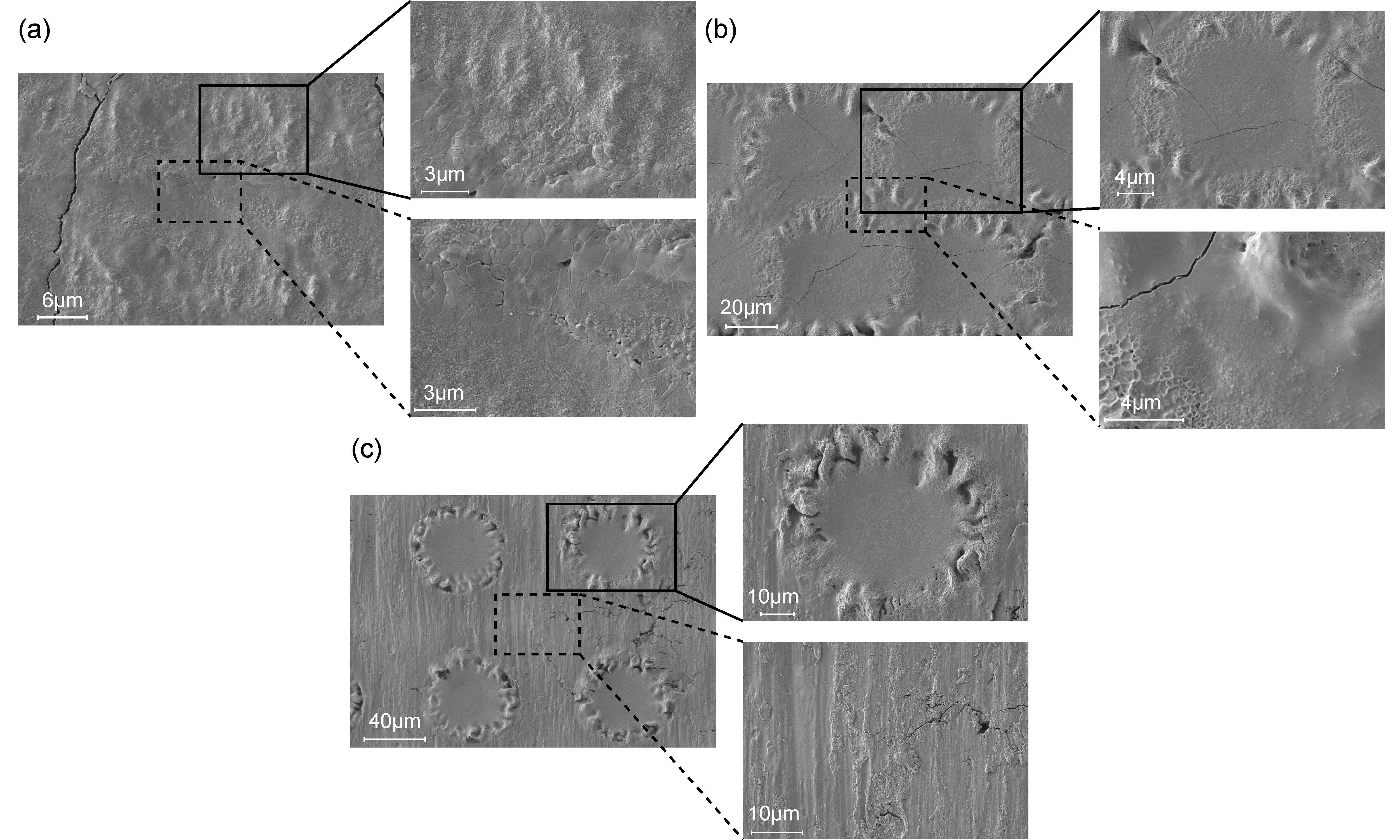


Fig.S4: (a) Local magnification of the SEM image of a sample with a point spacing of 20 μm, (b) 50 μm and (c) 100 μm.

To better observe the microscopic details of different regions, we captured high-magnification electron microscope images of the samples, as shown in Fig. 4(a)–(c). The images focus on specific spots within samples with spacings of 20 μm, 50 μm, and 100 μm. We also identified the geometric center of the four spots in each case.
